# Display of *E. coli* Alkaline Phosphatase pIII or pVIII Fusions on Phagemid Surfaces Reveals Monovalent Decoration with Active Molecules

**DOI:** 10.2174/1874091X00802010038

**Published:** 2008-04-15

**Authors:** Michael Weichel, Rolf Jaussi, Claudio Rhyner, Reto Crameri

**Affiliations:** 1 Swiss Institute of Allergy and Asthma Research (SIAF), Obere Strasse 22, CH-7270 Davos, Switzerland; 2 Biomolecular Research, Paul Scherrer Institut, CH-5232 Villigen PSI, Switzerland

## Abstract

Active alkaline phosphatase of *Escherichia coli* (PhoA, EC 3.1.3.1) was displayed *via *the leucine zipper element of the Jun-Fos heterodimer on the surface of filamentous phage and the kinetic parameters *K*_m_ and *k*_cat_ were determined. The *phoA* gene was cloned downstream of *fos* while *jun* was inserted upstream of *pIII* or *pVIII*, alternatively, in the pJuFo phagemid vector. Both fusion genes are regulated by independent *lacZ* promoters. PhoA displayed on the phagemid pIII surface exhibited a *K*_m_ of 11.2 µM with 4-nitrophenyl phosphate as substrate, which is consistent with data published for soluble PhoA. Based on these data we calculated the decoration of pJuFo phagemid with PhoA using the minor and major coat proteins pIII and pVIII as fusion partners under variable inducing conditions. We found that, even if the promoters are fully induced at a concentration of 1000 µM IPTG, the phagemids display maximally one copy of PhoA-Fos-Jun-coat protein fusion, irrespective of whether the protein is presented *via *pIII or pVIII. However, since PhoA is displayed in a native-like fashion, as deduced from the kinetic parameters of the enzymatic reaction, the pJuFo technology provides a versatile tool for the functional screening of complex cDNA libraries displayed on the phagemids' surface.

## INTRODUCTION

Most functional proteomic approaches aim at the identification of interactions between naturally occurring proteins. Once the interactions are known the resulting interactome networks can provide information about complex molecular interactions and improve our understanding of evolutionary, metabolic, molecular and cellular processes [1]. Recently, enhanced modifications of the original yeast two-hybrid system [2] have been used to generate entire interaction maps of the yeast *Saccharomyces cerevisiae* [3, 4] and the nematode worm *Caenorhabditis elegans* [5]. The problem of relatively frequent false-positive hints remains a major disadvantage of this *in vivo* technology [6]. As an alternative, lambda phage based screening or phage surface display of cDNA libraries are often favoured over the two-hybrid system, if a library needs to be screened against an immobilized monoclonal or oligoclonal ligand because both *in vitro* screening systems allow variation of the binding conditions.

Phage display differs from other screening systems because molecular libraries incorporated into the phage genome are expressed on the surface of phage particles [7]. The physical linkage between genotype and phenotype allows maintaining a phage library in the liquid phase and therefore efficient screening based on the power of affinity selection. cDNA phage display systems based on bacteriophages T7 [8, 9], lambda [10], and filamentous phage [11-15] have been recently reported and reviewed elsewhere [16, 17].

**Fig. (1):**
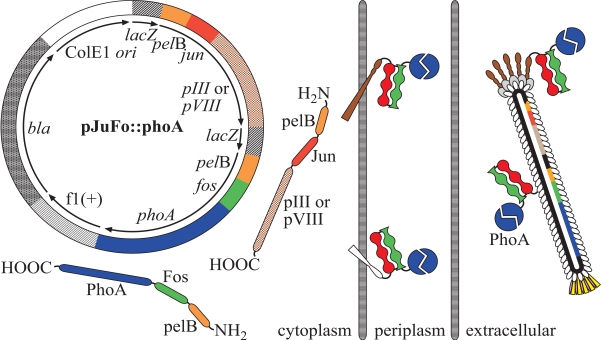
Genetic elements of the pJuFo vector and proposed mechanism for the assembly of PhoA-phagemids. In the present work PhoA was displayed either as pIII or pVIII fusion protein *via* the heterodimeric Jun-Fos linker on the surface of filamentous phagemid

Among the filamentous phage display approaches the pJuFo technology, which is based on an indirect fusion approach to display products on the surface of helper phage assisted phagemid assemblies, has widely been used for the surface expression of cDNA libraries, and yielded complex allergen repertoires of various allergenic sources [18]. Furthermore, the technology provided a valuable tool for the identification of tumor-associated antigens [19], self-antigens [20] or epitopes of polyclonal antibody sera [21]. Since the applications of this methodology are quite diverse, and yet it has not met substantial limitations, we investigated the efficiency of the pJuFo surface presentation choosing *E. coli* alkaline phosphatase (PhoA, EC 3.1.3.1) as a model system. *PhoA* was cloned adjacent to *fos* of the pJuFo vector and expressed as a Jun-Fos-pIII or Jun-Fos-pVIII fusion protein on the phagemids' surface (Fig. **1**). Under reaction conditions in which PhoA exhibits Michaelis-Menten behaviour we obtained the kinetic parameters *K*_m_ and *k*_cat_ of phagemid-displayed PhoA. Comparison of these kinetic parameters with those of wild-type PhoA allowed calculating the decoration of phagemid with functional PhoA fusion proteins.

## MATERIALS AND METHODS

### Construction of pJuFoVIII : : phoA

Production of phage displaying PhoA of *E. coli* as a pIII fusion protein by means of the Jun-Fos linker is described elsewhere [11, 37]. For the construction of a pVIII-display pJuFo vector, the DNA encoding pVIII was amplified by PCR using M13K07 helper phage as a template and the following oligonucleotides: 5^'^-primer pVIII: 5^'^-ACG GGA TCC GGT GGC GGT GGC TCT GCT GAG GGT GAC-3^'^, 3^'^-primer pVIII: 5^'^-GTG TTA CTA GCT AGC TTT AAT TGT ATC GGT TTA-3^'^. The PCR fragment was hydrolysed with *Bam*HI and *Nhe*I, gel purified and used to replace the *Bam*HI-*Nhe*I fragment in pJuFo. The ligation mixture was used to transform *E. coli* XL1-Blue (Stratagene, La Jolla, CA, USA) electrocompetent cells [38] which were then spread on LB agar plates with 100 µg/ml ampicillin and 10 µg/ml tetracycline (LB/Amp+Tc). Single colonies were picked and grown. DNA was prepared and the final construct was was verified by restriction analysis and DNA sequencing (Microsynth, Balgach, Switzerland). The pJuFoVIII:: phoA phagemid was constructed by *Xba*I+*Kpn*I hydrolysis of the pJuFoVIII vector and ligation with correspondingly hydrolysed phoA DNA derived from pJuFoIII::phoA [11].

### Preparation of PhoA-Phagemids

110-150 ml 2×YT medium with 100 µg/ml ampicillin and 10 µg/ml tetracycline (2×YT/Amp+Tc) were inoculated 1:50 with a stationary culture of *E. coli* XL1-Blue/pJuFo::phoA or XL1-Blue/pJuFoVIII::phoA and incubated at 37^°^C and 250 rpm until an OD_550_ of ≈ 0.5 was reached. Then helper phage VCS-M13 (Stratagene) was added at a multiplicity of infection (moi) of 10 and the culture was incubated for 30 min at 30^°^C, 150 rpm. After 30 min agitation of the culture was raised to 300 rpm and the culture was incubated for additional 9.5 h at 30^°^C. 2 h after infection with helper phage, kanamycin (kan) was added to a concentration of 70 µg/ml. For experiments, in which the decoration of phage with PhoA was determined in dependence of the inducer concentration, IPTG was added 30 min after infection with helper phage.

PhoA-phagemid containing supernatant was prepared 10 h after helper phage addition by centrifugation (4'500 × *g*, 4^°^C, 15 min) and PhoA-phagemid was precipitated by addition of ¼ volume 25% (w/v) polyethylene glycol (MW: 8'000), 15% (w/v) NaCl at 4^°^C for 20 min. After centrifugation (15'000 × *g*, 4^°^C, 20 min) the supernatant was removed and the precipitated phagemid was dissolved in 10 mM Tris/HCl pH 8.0 (4 vol. % of the original supernatant volume). After centrifugation (15'000 × *g*, 4^°^C, 10 min) the cleared solution was transferred into fresh tubes and PhoA-phagemid was precipitated again with PEG/NaCl as described above, the supernatant was removed quantitatively and precipitated PhoA-phagemid was dissolved in 10 mM Tris/HCl pH 8.0 (4 vol. % of the original supernatant volume). After final centrifugation (15'000 × *g*, 4^°^C, 10 min) the PhoA-phagemid solution was transferred into new tubes, kept at 4^°^C and readily used for the determination of infectivity and for enzymatic assays. Samples immediately not used for experiments were stored at -20^°^C directly after preparation.

### Inducibility of pJuFoIII: : phoA and pJuFoVIII : : phoA

To test the inducibility of the phagemid vectors by IPTG after addition of helper phage we prepared periplasmic extracts of XL1-Blue using a moderately modified protocol for cold osmotic shock [39]: portions of 25 ml were taken from a 200 ml PhoA-phagemid producing XL1-Blue culture at distinct times after VCS-M13 superinfection and centrifuged (4'500 × *g*, 4^°^C, 15 min). The supernatant was removed completely and bacterial pellets washed by gentle re-suspension in 1 ml of 2×YT followed by centrifugation (4'500 × *g*, 4^°^C, 15 min). After medium removal the bacteria were resuspended in 1 ml 500 mM Saccharose, 100 mM Tris/HCl, 1 mM EDTA pH 8.0 and incubated on ice for 30 min in the presence of 100 µg/ml hen egg white lysozyme (Roche, Basel, Switzerland). The spheroplasts were sedimented (15'000 × *g*, 4^°^C, 15 min) and the periplasmic fractions were stored at -20^°^C.

### Western Blots

Western blot analysis was used to detect wild-type (wt) PhoA (Sigma, St. Louis, MO, USA) or Fos-PhoA in the periplasmic fractions as well as in the phagemid preparations. Therefore, samples were subjected to SDS-PAGE (NuPAGE^™^, 12% Bis-Tris, Invitrogen^™^) and electrotransferred onto a Hybond^™^-P PVDF membrane (Amersham Pharmacia Biotech, Uppsala, Sweden). Free binding sites were blocked with 5% non-fat dried milk, 0.1 % (v/v) Tween-20 in TBS. PhoA was detected with horse radish peroxidase labelled rabbit polyclonal anti-bacterial AP mAb 7319 (1:10'000 in blocking buffer, Abcam, Cambridge, United Kingdom) and visualised by chemiluminescence (ECL^™^, Amersham Pharmacia Biotech) substrate.

### Enzymatic Assays

PhoA-phagemid enzyme activity was assayed basically as described by McCafferty *et al*. [31]. Briefly, reactions were initiated by adding 50 µl PhoA-phagemid solution to 950 µl of 4-nitrophenyl phosphate (Fluka, Buchs, Switzerland) at a range of concentrations in 1.052 M Tris/HCl pH 8.0. The initial rates were calculated from the change of the absorbance at 410 nm in the first 6 s of each reaction at 25^°^C using a molar absorption coefficient of 16'200 M^-1^ cm^-1^ [40]. The concentration of PhoA-phagemid was determined by preparing ssDNA using phenol/chloroform/ isopropanol extraction, ethanol precipitation and measuring at 260 nm. The A_260_/A_280_ ratio of the ssDNA prepared this way was always higher than 1.77. The molar absorption coefficients of the individual single stranded vector contructs were calculated with the Omiga 2.0 software (Oxford Molecular, Oxford, England). Purified *E. coli* PhoA was purchased from Sigma. The concentration was calculated by measuring the absorbance at 278 nm [41] using a *M*_r_ of 47'029 per subunit [28]. The kinetic parameters *K*_m_ and *k*_cat_ were derived fromnonlinear least-square fits according to the Michaelis-Menten model. For practical reasons *k*_cat_ values are expressed as molar activities (mol. act.) and specified in mol substrate/mol phage (or enzyme)/min throughout this paper. All absorption measurements were performed on an Uvikon XL spectrophotometer (Bio-Tek Instruments, Winooski, VT, USA).

### Phagemid Infectivity

Infectivity is defined as the ratio between infective and total phage. Total phagemid was measured by preparing ssDNA as described above, infective phagemid was determined by infecting exponentially growing *E. coli* XL1-Blue with three independent dilution series of each phagemid preparation and plating out on LB/Amp+Tc agar.

## RESULTS

### Kinetic Properties of PhoA Displayed on the Surface of pJuFo Phagemids

Display of cDNA libraries on the surface of filamentous phage and their screening for interacting proteins against immobilized targets still remains a challenge in proteomics. Although also pVI as fusion partner for cDNA products has been described [13, 14, 22], the pJuFo technology which uses the pIII-Jun-Fos-cDNA fusion is well established for the efficient selection and identification of protein-protein interacting partners [16]. To determine if these proteins are displayed in a native-like conformation, we have generated phagemid vectors displaying *E. coli* PhoA on the surface of filamentous phage particles as pIII or pVIII fusion proteins *via *the Jun-Fos linker, determined the kinetic parameters of the phagemid-displayed enzymes and compared them to those of soluble PhoA. It has been shown previously that functional PhoA can be displayed on the surface of filamentous phage [11, 14, 15, 23]. However, only McCafferty *et al. *[23] provide detailed kinetic data and show that PhoA directly fused to the N-terminus of pIII exhibits a different *K*_m_ compared to soluble PhoA.

As shown in Table **1** PhoA displayed on pJuFo –III phagemids exhibits a *K*_m_ of 11.2 µM, which is in agreement with published data of soluble PhoA [23, 24]. This value is independent from PhoA being displayed as pIII or pVIII fusion and independent of the IPTG concentration under which the nascent PhoA-phagemid is being produced.

Assuming that the individual rate constants composing *K*_m_ of PhoA-phagemid are comparable to those of soluble PhoA the number of active enzyme molecules displayed on each phagemid particle can be calculated. Fig. (**2**) shows the molar activities of wtPhoA, PhoA-pIII-phagemid and PhoA-pVIII-phagemid produced in the absence of IPTG. Under this condition PhoA is presented nearly twice as effectively *via *pIII than *via *pVIII. However, PhoA-pIII-phagemid shows a molar activity of 1587 mol substrate/mol phage/min compared to a molar activity of 3539 mol substrate/mol enzyme/min for soluble PhoA, indicating that only 45% of the phagemids display one active enzyme molecule *via *pIII.

Another aim of this study was to investigate if and to what extend addition of IPTG during phagemid production would increase the decoration of phagemids with active enzyme. We therefore tested in a first step the inducibility of the pJuFo: : phoA vectors by preparing periplasmic fractions from phagemid producing XL1-Blue cultures grown with or without 1 mM IPTG. The periplasmic fractions were then subjected to Western blot analysis and the Fos-PhoA fusion protein was detected by HRP-labelled anti-bacterial PhoA mAb 7319. As shown in Fig. (**3**), induction of *fos: : phoA* expression is enhanced with IPTG in either of the phagemid vectors during the entire period of phage propagation. Surprisingly, the difference in *fos::phoA* expression with and without IPTG is much less pronounced in pJuFoIII::phoA (Fig. **3A**) compared to pJuFoVIII: : phoA (Fig. **3B**). This difference might be more obvious if we consider that the periplasmic extracts of the cultures grown in medium with 1 mM IPTG were prepared from a 10 to 15% reduced cell number compared to those grown without IPTG (Fig. **3C**).

To directly investigate the effect of the inducer on the valency of PhoA-phagemids we then added IPTG to final concentrations of 1 µM, 10 µM, 100 µM and 1000 µM 30 min after 10 µM, 100 µM and 1000 µM 30 min after infecting the bacteria with helper phage and prepared the phagemids after another 9½ h. The molar activities of these PhoA-phagemids are shown in Fig. (**4**). We observed a twofold increase of PhoA-pVIII-phagemid activity in the range between 1 µM and 10 µM IPTG with an apparent activity saturation at IPTG concentrations higher than 10 µM. An increase in molar activity could also be detected in the case of PhoA-pIII-phagemids at IPTG concentrations ≥ 10 µM, but it was much less pronounced. Because both *jun: : gIII* and *fos: : phoA* are under control of *lacZ* promotors, we consider this behaviour to reflect the *in vivo* dissociation constant of inducer and Lac repressor, which is 5.7 µM [25].

The finding that a maximum of one copy of PhoA is displayed on either species of pJuFo phagemids could also be confirmed by Western blot analysis (Fig. **5**).

However, it should be annotated that both total phagemid yield as well as their infectivity decrease with increasing IPTG concentration as shown in Fig. (**6**).

The titer of PhoA-pIII-phagemids produced at IPTG concentrations ≥ 10 µM was less than 50 % compared to the phagemid titers obtained without induction, while infectivity decreased almost twofold from 1.8 to 1.0 percent. For PhoA-pVIII-phagemids the total yield even dropped below 30 % at IPTG concentrations ≥ 10 µM, while infectivity decreased from 5.5 (no IPTG) to 3.3 percent (1000 µM IPTG) and thus was always higher compared to the pIII fusion. However, infectivity of PhoA-pVIII-phagemids decreased significantly after 48 h storage at 4^°^C, indicating that the phage displaying the PhoA-pVIII fusion protein is less stable than the phage displaying the PhoA-pIII fusion protein (data not shown). Therefore we recommend the use of pIII as a fusion partner for phage surface display of cDNA libraries.

## DISCUSSION

Success and failure of phage display methods critically depend on distinct experimental variables and parameters, as was indicated on the basis of stochastic models [26]. Besides the effective concentration of immobilized high affinity ligands and the stringency of washing steps, particularly complexity and titer of a phage library as well as valency of phage have an impact on the outcoming of a biopanning-based selection experiment. In order to investigate valency, infectivity and display quality of the pJuFo phagemid, which has proven a versatile vector for the screening of cDNA libraries of various allergic sources [18], human tumor cells [19], mice B cells [27], and human fibroblasts [20], we generated pJuFo-PhoA phagemids in which PhoA was either fused to the minor coat protein pIII or to the major coat protein pVIII *via *a Jun-Fos linker. PhoA is a homodimeric protein located in the periplasm of *E. coli* and has a molar mass of 47 kDa per subunit [28]. There is strong evidence from the literature that only dimeric PhoA is catalytically active [29, 30]. Therefore, it was surprising that the Michaelis constant *K*_m_ of the phage-displayed PhoA was identical to that of soluble PhoA (Table **1**), indicating that dimeric proteins can be displayed in a native-like fashion on the surface of pJuFo phagemids and that the two intramolecular disulfide bonds present in the PhoA subunit are formed correctly. Furthermore, we may conclude that the individual rate constants composing *K*_m_ in the PhoA-phagemids are identical to those of soluble PhoA. Thus, *k*_cat_ or molar activities of PhoA-phagemids and soluble PhoA may be compared directly to calculate the valency of PhoA-phagemids.

As shown in Fig. (**4**) all of the PhoA-phagemid populations prepared from incubations with or without IPTG display lower activity than the soluble PhoA indicating that statistically maximally one of the five [31] copies of pIII and one of 2700 copies of pVIII represents a PhoA-coat protein fusion. This observation is supported by Western blot analysis (Fig. **5**), and thus we may conclude that the majority of the displayed PhoA fusion molecules are also enzymatically active.

**Fig. (2):**
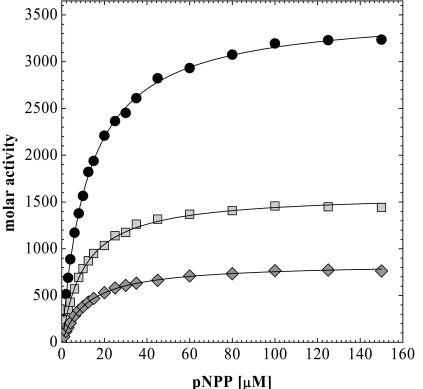
Michaelis-Menten plot of soluble PhoA (solid black circles), PhoA-pIII-phagemid (light grey squares) and PhoA-pVIII-phagemid (dark grey diamonds). Molar activities are plotted against the substrate concentration to calculate the kinetic parameters summarized in Table 1

**Fig. (3):**
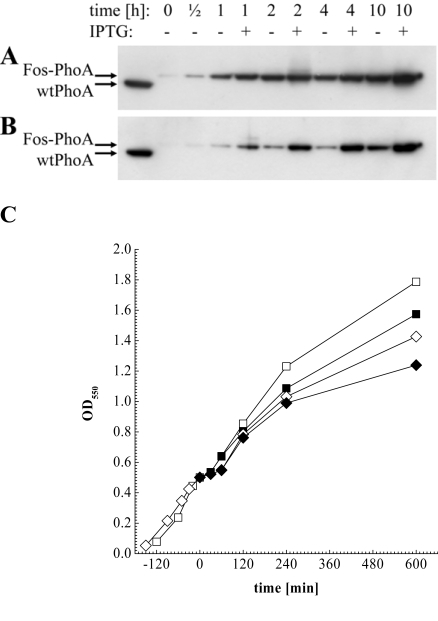
Western blot analysis of periplasmic extracts directly before, ½ h, 1 h, 2 h, 4 h and 10 h after helper phage superinfection, and of soluble wtPhoA (left lane). (A) Fos-PhoA detected in XL1-Blue harbouring pJuFoIII::phoA without (-) and in the presence of 1 mM IPTG (+). (B) Fos-PhoA detected in XL1-Blue harbouring pJuFoVIII::phoA grown without (-) and in the presence of 1 mM IPTG (+). (C) Growth rate of XL1-blue/pJuFoIII::phoA (squares) and XL1-blue/pJuFoVIII::phoA (circles) grown without (open symbols) and with 1 mM IPTG (solid symbols)

**Fig. (5):**
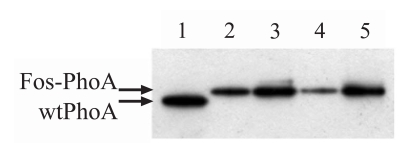
Western blot analysis of 10^12^ phage particles (quantified by A_260_ measurements) prepared 10 h after helper phage infection and detection by polyclonal anti-PhoA rabbit antibody ab7319. Lane 1: 7.81∙10^-5^ mg wtPhoA (= 10^12^ PhoA subunits). Lane 2: PhoA-pIII-phagemid amplified without IPTG. Lane 3: PhoA-pIII-phagemid amplified in the presence of 1 mM IPTG. Lane 4: PhoA-pVIII-phagemid amplified without IPTG. Lane 3: PhoA-pVIII-phagemid amplified in the presence of 1 mM IPTG.

It has been shown earlier that the decoration of filamentous hybrid phage with peptides fused to pVIII critically depends on the length of the peptide [32-34] and, even more, on the rate of processing of the molar pro-coat fusion protein [35] which is a critical step in recombinant phage assembly. As a result the infectivity of phage populations that display a 10 or 16 amino acid peptide drops from 20% to 1% [34], respectively. Malik *et al.* [40] have impressively demonstrated by Western blot analysis that 12mers and 16mers can be displayed in up to 40% and 25% of the pVIII copies, respectively. According to their model-building approach the phage coat could even be decorated with a folded 100 kDa protein at a saturation of 24% [35]. However, experiments in which the display of a scFv *via *pIII and pVIII was directly compared by ELISA clearly showed that pIII was more efficient than pVIII in displaying coat fusion proteins even though there are much less copies of pIII present in a phage particle [36]. Those results generally corroborate our findings that the display of PhoA *via *pIII is more efficient than *via *pVIII when the PhoA-coat protein fusion is produced from a phagemid vector in the absence of induction (Fig. **2**). But so far there is no hint in the literature why the copy number of displayed PhoA should be restricted to one per phage particle, even when fused to the major coat protein pVIII.

When produced in the presence of IPTG as strong inducer of the *lacZ* promoter, the titer of PhoA-pVIII-phagemids was always lower compared to PhoA-pIII-phagemids. However, the infectivity of the PhoA-pVIII-phagemid remained higher than that of the PhoA-pIII-phagemid.

Taken into account that the complexity of a primary cDNA library is usually in the range of 10^7^ primary clones and that the amplified phagemid populations from all experiments exceeded a titer of 1·10^11^ transforming units per ml (tu/ml) it remains irrelevant whether the cDNA products are displayed *via *pIII or pVIII. In any case the cDNA library will be entirely displayed on the phage surface and makes pJuFo an ideal phagemid vector for the screening of cDNA libraries from various origins.

## Figures and Tables

**Fig. (4):**
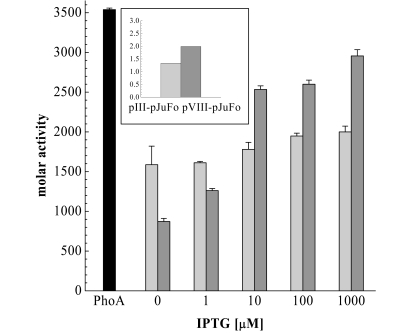
Molar activities of soluble dimeric PhoA (black), PhoA-pIII-phagemid (light grey) and PhoA-pVIII-phagemid (dark grey) in dependence on the IPTG concentration. Molar activities of PhoA-phagemid are measured per phage particle. As control pIII-pJuFo and pVIII-pJuFo phagemids without a PhoA fusion were used. The molar activities of these control samples were 1.33 and 1.99 mol substrate converted/mol phage/min respectively (small figure top left)

**Fig. (6):**
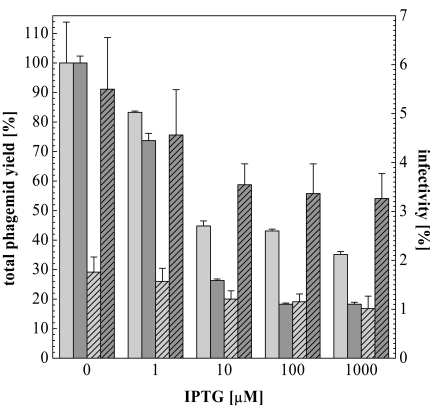
Phage yields (solid bars), and infectivities (diagonals) of PhoA-pIII-phagemid (light grey) and PhoA-pVIII-phagemid (dark grey) prepa-rations in the absence and presence of IPTG during phage production. 100% represent 4·1013 phagemids/ml (PhoA-pIII-phagemid) and 9·10^13^ phagemids/ml (PhoA-pIII-phagemid) respectively. Infectivity is measured in transforming units per ml.

**Table 1.** Michaelis Constants of Different Phagemid Prepara-tions and of Soluble PhoA

Sample	c(IPTG) [µM]	***K*_m_ [µM]^d)^**
pIII-PhoA	0	11.2 (± 0.1)
pIII-PhoA	1	12.3 (± 0.9 )
pIII-PhoA	10	9.0 (± 0.8 )
pIII-PhoA	100	9.8 (± 1.1 )
pIII-PhoA	1000	10.1 (± 1.2 )
pVIII-PhoA	0	12.9 (± 1.0 )
pVIII-PhoA	1	12.9 (± 1.2 )
pVIII-PhoA	10	12.3 (± 0.9)
pVIII-PhoA	100	11.0 (± 0.7)
pVIII-PhoA	1000	11.0 (± 0.6)
PhoA^ a)^		12.3 (± 0.2)
PhoA^b)^		12.7
PhoA^c)^		8.5

^a)^*Km* from the present work, ^b)^*Km* from [ 24], ^c)^*Km* from [ 23]. ^d)^The standard deviation derived from three experiments performed with independent freshly prepared phagemids is given in parentheses
